# Application of Electron Paramagnetic Resonance Spectroscopy for Validation of the Novel (AN+DN) Solvent Polarity Scale

**DOI:** 10.3990/ijms9071321

**Published:** 2008-07-17

**Authors:** Luciana Malavolta, Erick F. Poletti, Elias H. Silva, Shirley Schreier, Clovis R. Nakaie

**Affiliations:** 1 Department of Biophysics, Universidade Federal de Sao Paulo (Unifesp), Rua 3 de Maio 100, CEP 04044-020, São Paulo, SP, Brazil. E-Mails: luciana@biofis.epm.br (L. M.); erick@biofis.epm.br (E. P.); ehsilva@biofis.epm.br (E. S.); 2 Department of Biochemistry, Institute of Chemistry, Universidade de Sao Paulo, Sao Paulo, Brazil. E-Mail: schreier@iq.usp.br (S. S.); 3 Instituto Israelita de Ensino e Pesquisa Albert Einstein, Sao Paulo, SP, Brazil

**Keywords:** polarity, solvent, EPR, spin label, isotropic hyperfine splitting constant

## Abstract

Based on solvation studies of polymers, the sum (1:1) of the electron acceptor (AN) and electron donor (DN) values of solvents has been proposed as an alternative polarity scale. To test this, the electron paramagnetic resonance isotropic hyperfine splitting constant, a parameter known to be dependent on the polarity/proticity of the medium, was correlated with the (AN+DN) term using three paramagnetic probes. The linear regression coefficient calculated for 15 different solvents was approximately 0.9, quite similar to those of other well-known polarity parameters, attesting to the validity of the (AN+DN) term as a novel “two-parameter” solvent polarity scale.

## 1. Introduction

No single solvent polarity scale has yet been accepted as being the most appropriate for interpreting solvent effects. The findings obtained to date only underscore the difficulties in attaining a consensus regarding the rules that might govern solute-solvent interactions. A great number of empirical polarity scales based on experimental studies have been proposed, most of which employed single solute-models in order to investigate (spectroscopically, thermodynamically, kinetically, etc.) the interaction between solutes and solvents of different polarities [1, 2].

In a conceptual departure from most prior efforts to define an accurate polarity term, we previously proposed an alternative polarity scale based on experiments designed to assess solvation of model resins and peptide-resin (microscopic measurement of bead swelling) in solvent systems broadly encompassing the polarity scale [3, 4]. In these works, we have hypothesized that the 1:1 sum of Gutman's [5] electron acceptor number (AN) and electron donor number (DN) numbers would be a dimensionless, accurate polarity term. Due to the presence of opposing concepts within the same parameter, the (AN+DN) term was denoted an *amphoteric* constant or scale [6]. The application of this novel polarity constant can facilitate the prediction of polymer or peptide-polymer solvation, thereby improving techniques such as solid-phase peptide synthesis [7–9]. In addition, the acquisition of specific knowledge regarding this type of solute-solvent interaction process has been of great value for the development of advantageous experimental approaches in the field [10–12]. Furthermore, the combination of the antagonic AN and DN concepts has also been extremely useful for understanding certain rules that govern solubilization of intractable and strong aggregate solutes [13, 14], including peptides responsible for the development of neurodegenerative disorders such as Alzheimer's disease [15, 16].

In the interest of continuously evaluating the validity of the newly-introduced (AN+DN) scale, the present study of solvent polarity evaluates the well-known dependence of the electron paramagnetic resonance (EPR) isotropic hyperfine splitting constant (a_N_) on the polarity of the medium. This spectral parameter is strongly affected by the proticity and therefore by the polarity of the medium as a consequence of the proton acceptor nitroxyl group containing the unpaired electron of the paramagnetic molecules [17–20]. Greater polarity or proticity of the medium results in higher a_N_ values, since the unpaired electron is induced to localize more closely to certain atoms, such as the nitrogen atom of the N-O moiety of the probe.

**Figure 1.**
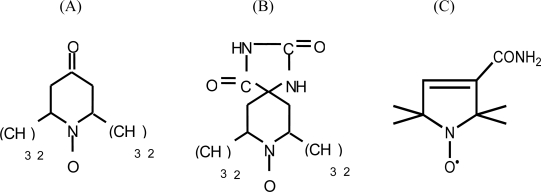
Structures of (A) 2,2,6,6-tetramethyl-4-piperidone-1-oxide; (B) 5-4-hydantoin-2,2,6,6-tetramethyl-4-piperidone-1-oxide; and (C) 3-carbamoyl-2,2,5,5-tetramethyl-3-pyrrolin-1-yloxy.

From among the different types of paramagnetic probes, the following nitroxide-radical spin labels were selected for the present study: (A) 2,2,6,6-tetramethyl-4-piperidone-1-oxide; (B) 5,4-hydantoin-2,2,6,6-tetramethyl-4-piperidone-1-oxide and (C) 3-carbamoyl-2,2,5,5-tetramethyl-3-pyrrolin-1-yloxy (Figure 1). Spin labels (A) and (B) are precursors of the amino acid-type spin probes 2,2,6,6-tetramethylpiperidine-1-oxyl-4-amino-4-carboxylic acid (TOAC) and 2,2,5,5-tetramethylpyrrolidine-1-oxyl-3-amino-4-carboxylic acid (POAC) [21, 22], both further chemically derived for allowing their pioneering insertion within a peptide sequence [23–25]. The EPR spectra of these three spin probes were recorded for 15 single solvents, and binary correlations were drawn between the a_N_ values and the (AN+DN) terms. For comparison, binary correlations were also drawn between the a_N_ values and other known polarity scales. Among these, the Dimroth-Reichardt E_T_(30) parameter [26], the Hildebrand solubility term (δ) [27] and the dielectric constant ε [28] were selected. Gutman's AN and DN numbers [5] were also examined in isolation within this polarity-a_N_ relationship.

## 2. Results and Discussion

The three spin probes used as models for the present study (Figure 1) were dissolved at a final concentration of 5 × 10^−5^ M in order to avoid saturation effects in the solvents listed in Table 1 below. This Table also presents the AN, DN, (AN+DN), E_T_(30), δ and ε values found in the literature for the 15 solvents listed. The first two solvent parameters were added because some authors have considered them to be polarity parameters [29].

**Figure 2.**
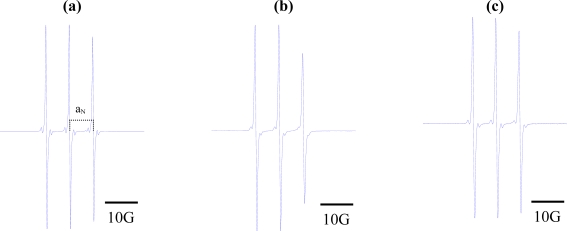
Electron paramagnetic resonance spectra of (a) 2,2,6,6-tetramethyl-4-piperidone-1-oxide; (b) 5,4-hydantoin (2,2,6,6-tetramethyl-4-piperidone-1-oxide; and (c) 3-carbamoyl-2,2,5,5-tetramethyl-3-pyrrolin-1-yloxy. Concentration of samples: 5 × 10^−5^ M of each component in dimethyl sulfoxide.

The EPR spectra of the spin label in these solvents were acquired at 9.5 GHz, and a_N_ values were determined by measuring the distance (in Gauss) between the h_+1_ and h_0_ EPR peaks. Figure 2 displays the EPR spectra of the three paramagnetic probes in dimethyl sulfoxide. As expected, narrow lines were observed, reflecting the high level of activity of these small molecules in isotropic medium [30].

**Figure 3.**
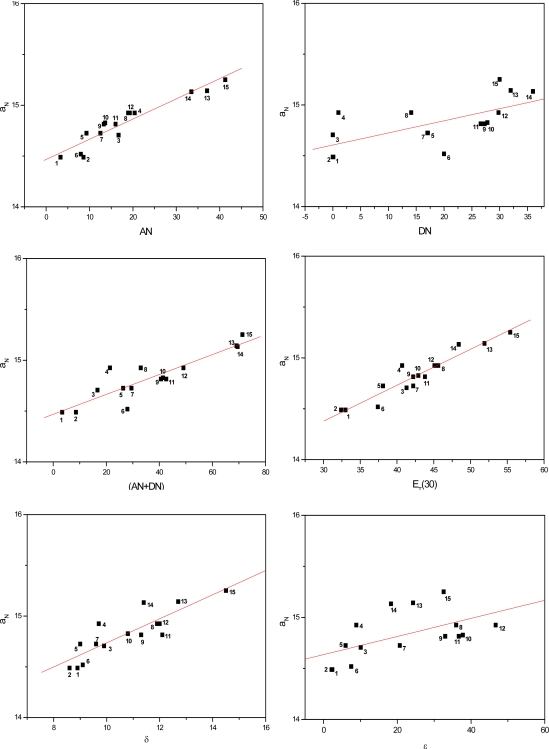
Values of a_N_ of nitroxide (A) as a function of solvent AN, DN, (AN+DN), E_T_30, δ and ε.

**Figure 4.**
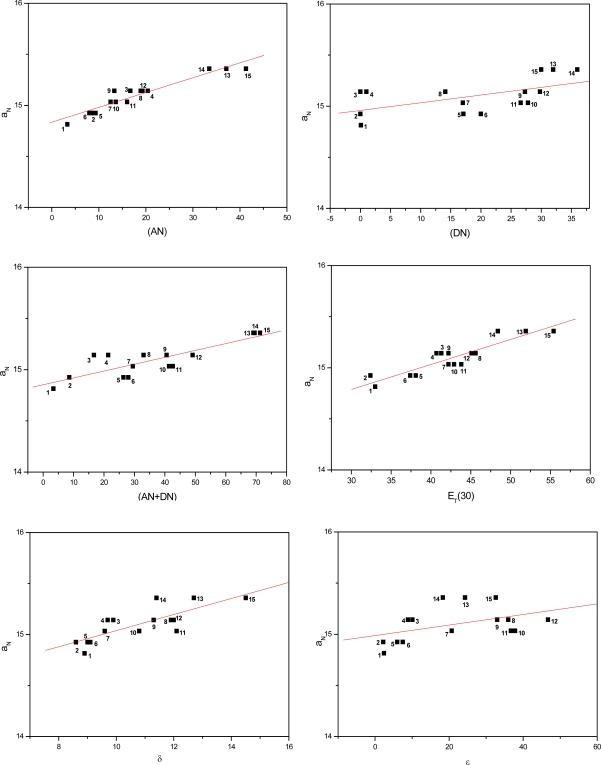
Values of a_N_ of nitroxide (B) as a function of solvent AN, DN, (AN+DN), E_T_30, δ and ε.

**Figure 5.**
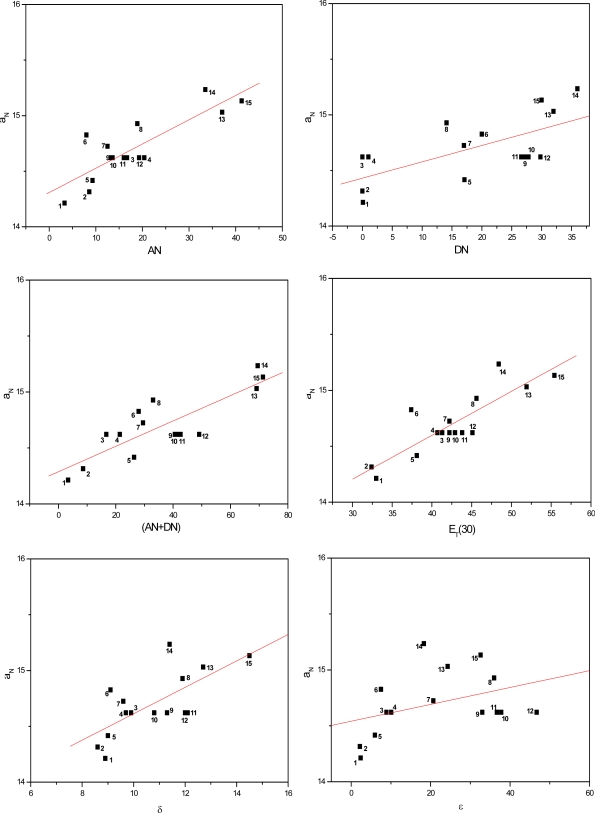
Values of a_N_ of nitroxide (C) as a function of solvent AN, DN, (AN+DN), E_T_30, δ and ε.

Figures 3 to 5 show the correlations found between the a_N_ values of probes in the solvents listed in Table 1 and their AN, DN, (AN+DN), E_T_(30), δ and ε values. It can be seen that the linear correlations were of different degrees, depending on the solvent term and on the type of paramagnetic probe.

To determine which solvent property presented a stronger linear correlation with the a_N_ parameter, a comparative study was conducted (Table 2). The average linear regression coefficients (r) were calculated for all conditions. Among the solvent parameters, the AN, E_T_(30) and (AN+DN) presented the strongest linear correlations, followed by δ, DN and the dielectric constant ε.

It is noteworthy that, in agreement with the results of earlier investigations with polymers, the dielectric constant presented the weakest correlation with the polarity of the medium. This finding with this solvent parameter is indeed expected as according to several reports in the literature, it is dependent on the class of solvent [31–33]. The dielectric constant varies when the solvent form or not hydrogen bonds and/or van der Waals interactions with the N-O moiety of the spin probes, inducing redistribution of the electrons in the medium. This is the case of alcohols such as methanol and propanol in the first class of solvent and ethyl acetate and acetone in the second. In the latter case, a weaker attraction between the Lewis base N-O groups of the spin probe and permanent or induced dipoles of the apolar solvent molecules may occur [19]. Nevertheless, due to the small amount of solvent systems assayed in this study, it was not possible to distinguish (in Figures 3 to 5), characteristic curves for each class of solvents (polar or apolar).

**Scheme 1.**
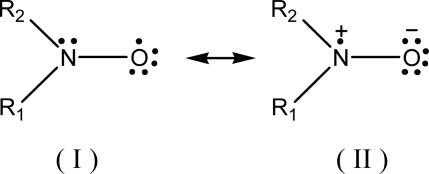
Canonical structures of the nitroxide-free radical.

The relationship between ESR spectral a_N_ parameter and the (AN+DN) or any other polarity or some other physicochemical terms might be also alternatively investigated based on some empirical molecular computational methods [19, 31]. In this case, it must be first considered the existence of the two canonical structures of the nitroxyl group, shown in Scheme 1:

The difference between these two forms is based upon the localization of the unpaired electron. The structure (II) localizes this electron on the N atom (for instance, in the case of nitroxide-bearing probe), and is responsible for the ESR three-line ^14^N hyperfine splitting. This occurs with more polar/hydrogen donor-type solvents that tend to attract the electronic pair to the atom of oxygen, increasing the a_N_ values. Opposite process occurs when in less polar solvents (structure I).

Besides these specific features of the N-O moiety, some aspects of the two π-molecular orbitals, composed from combination of the 2p_x_ atomic orbitals (AO) of N and O atoms must be also taken into account. Among the seven electrons of the canonic structure (I), two are in the N 1s orbital, three associated with the hybridized σ molecular orbital of the R1-N, R2-N and N-O bonds. The remaining two electrons are in the 2p_z_ π orbital. Otherwise, the eight electrons of oxygen atom are distributed as follows: two in the 1s AO, one in the 2p_z_ π orbital, a lone pair in the 2s AO, one in the 2p_y_ AO and finally, one in the 2p_x_σ MO of the N-O bond. Thus, for the two-center π-system, N and O atoms contribute with two and one electrons, respectively [31].

Regardless of the molecular orbital method to be applied, these N-O orbital details must be primary considered in combination with some experimental data collected in each type of approach tested. Nevertheless, besides the mentioned works (references [19] and [31]), there are others to be accessed for further examining the relationship between the a_N_ and solvent polarity terms through the molecular orbital calculation theories [34–36].

In addition, one must remember that the relationship between EPR a_N_ parameter and the polarity of the medium examined herein is valid. The early proposed definition of polarity as simply the solvent′s overall solvation capability (or solvation power) [19, 37] was officially accepted by the IUPAC committee [38]. In other words, different types of molecular association involving the spin probe and solvent molecules such as hydrogen bonding, van der Waals interaction or any type of electron redistribution in the vicinity of the N-O moiety are all, included in the polarity definition.

In summary, results of the present study confirm that the recently proposed (AN+DN) constant seems to fulfill the requisites to be considered an alternative polarity scale. This assertion has been verified in solvent effect studies involving solvation of dozens of polymers and peptide-polymers with different polarity characteristics in nearly 30 single and mixed solvent systems [3, 4].

It is of note that the dimensionless (AN+DN) scale, which ranges from zero (toluene) to almost 130 (trifluoromethanesulfonic acid) [4], has other unique advantage in terms of elucidating the solute solvation effect of solvents. The experiments detailed in a number of studies depict several examples of self-neutralizing effects occurring, for instance, when strong electrophilic trifluoroethanol, hexafluoroisopropanol or water are mixed with strong nucleophilic solvents such as dimethylsulfoxide [3, 4, 13, 14]. In such cases, one component trends to interact with its amphoteric counterpart and does not solvate the aggregated solute molecules, inducing their dissociation. This process also occurs in single solvents such as acetonitrile, acetone or isopropanol, which have quite similar electrophilicity and nucleophilicity characteristics (similar AN and DN values), inhibiting their capacity to disaggregate, for instance, peptide chains attached to a polymeric matrix or even free in solution. None of the single-term polarity scales, such as AN, E_T_(30), δ or ε, can explain these types of important solute-solvent interactions.

## 3. Experimental Section

### 3.1. Materials

The 3-carbamoyl-2,2,5,5-tetramemtyl-3-pyrrolin-1-yloxy used for the synthesis of the POAC spin label was acquired from Aldrich Co. The 2,2,6,6-tetramethyl-4-piperidone-1-oxide and 5-4-hydantoin-2,2,6,6-tetramethyl-4-piperidone-1-oxide, both intermediates of the synthesis of the TOAC probe, were obtained as previously described [21, 22]. The solvents were all of analytical grade, were all acquired from different sources and all met American Chemical Society standards.

### 3.2. Methods

The EPR spectra were obtained at 9.5 GHz with a Varian E-4 spectrometer at room temperature (22±2 °C), using flat quartz cells for liquid solutions (James Scalon, Costa Mesa, CA, USA). The magnetic field was modulated with amplitudes smaller than one-fifth of the linewidths, and the microwave power was set to 5 mW in order to avoid saturation effects. The spin probe solution was 5 × 10^−5^ M. Estimated uncertainties are ±0.03 G for a_N_ values.

## Figures and Tables

**Table 1.** Solvent Parameters.

	Solvent	AN^a^	DN^a^	(AN+DN)^b^	E_T_(30)^c^ (kcal/mol)	δ^d^ (cal/mL)^1/2^	ε^e^
1	Toluene	3.3	0.1	3.4	33.0	8.9	2.4
2	Carbon tetrachloride	8.6	0.0	8.6	32.4	8.6	2.2
3	1,2-Dichloroethane	16.7	0.0	16.7	41.3	9.9	10.1
4	DCM	20.4	1.0	21.4	40.7	9.7	8.9
5	Ethyl acetate	9.3	17.1	26.4	38.1	9.0	6.0
6	THF	8.0	20.0	28.0	37.4	9.1	7.5
7	Acetone	12.5	17.0	29.5	42.2	9.6	20.7
8	Acetonitrile	18.9	14.1	33.0	45.6	11.9	36.0
9	NMP	13.3	27.3	40.6	42.2	11.3	33.0
10	Dimethylacetamide	13.6	27.8	41.4	42.9	10.8	37.8
11	DMF	16.0	26.6	42.6	43.8	12.1	36.7
12	DMSO	19.3	29.8	49.1	45.1	12.0	46.7
13	EtOH	37.1	32.0	69.1	51.9	12.7	24.3
14	Propanol	33.5	36.0	69.5	48.4	11.4	18.3
15	MeOH	41.3	30.0	71.3	55.4	14.5	32.6

DCM: dichloromethane;

THF: tetrahydrofuran;

NMP: methylpyrrolidone;

DMF: dimethylformamide;

DMSO: dimethyl sulfoxide;

EtOH: ethanol;

MeOH: methanol

a Ref. 29;

b Refs. 3–4;

c Ref. 26;

d Ref. 27;

e Ref. 28

**Table 2.** Correlations between solvent parameters and a_N_ values.

Parameter	Linear regression coefficient (r)			
	A	B	C	(x̄)^a^
AN	0.94	0.95	0.84	0.91
DN	0.66	0.58	0.66	0.63
(AN+DN)	0.89	0.85	0.84	0.86
E_T_(30)	0.95	0.91	0.86	0,91
δ	0.86	0.79	0.70	0.78
ε	0.56	0.45	0.39	0.47

A: 2,2,6,6-tetramethyl–4-piperidone-1-oxide;

B: 5–4-hydantoin-2,2,6,6-tetramethyl–4-piperidone-1-oxide;

C: 3-carbamoyl-2,2,5,5-tetramethyl-3-pyrrolin-1-yloxy;

a Average of linear regression coefficient values.
